# Evaluating the effectiveness of chemotherapy for thymic epithelial tumors using the CD‐DST method

**DOI:** 10.1111/1759-7714.13362

**Published:** 2020-03-20

**Authors:** Lei Yu, Bao‐xun Zhang, Xin Du, Zhen Yu, Xing‐guo Yang, Yu‐xuan Jiang

**Affiliations:** ^1^ Department of Thoracic Surgery, Beijing Tongren Hospital Capital Medical University Beijing China

**Keywords:** CD‐DST, chemotherapy, sensitivity, TETs

## Abstract

**Background:**

Thymic epithelial tumors (TET) are frequently eligible for curative‐intent surgical resection. For locally advanced TETs, chemotherapy has been used to both reduce the tumor burden and achieve prolonged disease control. However, effective therapy for this disease largely remains to be determined. Here, we report the chemosensitivity of 100 patients with TETs determined by the collagen gel droplet embedded culture‐drug sensitivity test (CD‐DST).

**Methods:**

A total of 100 patients with TETs underwent surgical resection. The efficacy of antitumor agents on TET cells was tested by CD‐DST.

**Results:**

Thymic epithelial tumors were pathologically confirmed after surgery: two cases were type A thymoma, 17 were type AB, 12 were type B1, 44 were type B2, 12 were type B3, and there were 13 cases with thymic carcinoma. A total of 36% patients with TETs were sensitive to different types of chemotherapeutic agents. There was no significant differences in age, histological type, clinical staging, or association with autoimmune diseases between sensitive and nonsensitive cases. Type B1 and B2 thymoma were relatively more sensitive to chemotherapeutic agents (6/12 and 18/44, respectively), while sensitivity of type B3 cases to chemotherapeutic agents was much lower (only 2/12). Cases with type A thymoma were not sensitive to any antitumor drugs. Among 11 chemotherapeutic agents tested in our study, the sensitivity of TETs to EPI was the highest (16%). No patients with thymoma were sensitive to Alimta (Pemetrexed).

**Conclusions:**

Our work illuminates the effectiveness of chemotherapy for TETs and provides important clues for choosing antitumor drugs with relatively high drug sensitivity to TETs in advance.

## Introduction

Thymic epithelial tumors (TETs) represent the most common anterior mediastinal compartment neoplasm, originating from the epithelial cell population in the thymus. Due to their different histological types, TETs show different clinical characteristics. Furthermore, TETs are often associated with autoimmune disorders,^1^, ^2^, ^3^ the most common being myasthenia gravis (MG). Because the tumorigenesis of TETs still remains unknown, there is a lack of effective molecularly targeted therapies to treat thymoma.^4^, ^5^ TETs are frequently eligible for upfront curative‐intent surgical resection. For some cases with a locally advanced TET at the time of diagnosis, with invasion of neighboring organs, dissemination to the pleura, pericardium, or less frequently extra‐thoracic organs, chemotherapy has been used both to reduce the tumor burden ‐ possibly allowing subsequent surgery and/or radiotherapy ‐ and to achieve prolonged disease control. However, effective therapy for this disease largely remains to be determined. Some anticancer drugs have been reported to be effective in some cases of thymoma or thymic carcinoma.^6^, ^7^, ^8^, ^9^ Unfortunately, there is no single drug which shows a high clinical response. No systematic research has demonstrated the exact thymic tumor responses to chemotherapy. Former studies have indicated that the collagen gel droplet embedded culture‐drug sensitivity test (CD‐DST)^10^ could approximate the clinical effect in different types of malignant tumors^11^, ^12^ and have a high predictive accuracy for response to chemotherapy, reaching up to 84.1%.^10^ In this study, we report on the chemosensitivity of 100 patients with TETs by CD‐DST.

## Methods

### Patients

Between 2015 and 2018, 100 patients with TETs underwent surgical resection at Beijing Tongren hospital. There were 43 (43%) female and 57 (57%) male patients whose mean age was 47 years (ranging from 27 to 82 years) and they had not been treated with chemotherapy before surgery. The clinical profiles of the 100 patients are summarized in Table 1. Among the patients with TETs, 67 had autoimmune diseases (such as MG, primary adrenocortical hypofunction, optic neuritis, optic nerve degeneration, allergic dermatitis, Lupus erythematosus, etc), while 33 had no evidence of autoimmune disease. These patients underwent extended thymectomy using the trans‐sternal approach (*n* = 27) or video‐assisted thoracoscopic surgery (VATS) (*n* = 73). For patients with tumor nodules found on the pleural surface, cytoreductive surgery was performed.

**Table 1:** Patient characteristics

	Total patients	Sensitive cases	Nonsensitive cases	*P*‐value
Number	100	36	64	
Median age (range, years)	47 (27–82)	48 (32–72)	47 (27–82)	1.000
Male (*n*)	57	19	38	0.536
Female (*n*)	43	17	26	
WHO histological type				
A	2	0	2	0.581
AB	17	6	11	
B1	12	6	6	
B2	44	18	26	
B3	12	2	10	
Thymic carcinoma	13	4	9	
Masaoka's clinical staging				
I	25	10	15	0.451
II	41	12	29	
III	21	10	11	
IV	13	4	9	
Associated with autoimmune diseases (cases with MG)	67 (61)	27 (24)	40 (37)	0.269
Without any autoimmune disease	33	9	24	

### Tumor tissue samples

Samples of TETs were collected from fresh specimens during surgical procedures at Beijing Tongren Hospital. All TETs were reclassified according to the WHO histologic classification^13^ and the Masaoka clinical staging system.^14^

Antitumor agents included 5‐furuolouracil (5FU, 1.0 μg/mL), Epirubicin (EPI, 0.1 μg/mL), adriamycin (ADR, 0.02 μg/mL), cisplatin (CDDP, 0.2 μg/mL), carboplatin (CBDCA, 2.0 μg/mL), Etoposide (VP‐16, 1.0 μg/mL), Paclitaxel (PAC, 1.0 μg/mL), Navelbine (VNR, 0.01 μg/mL), Gemcitabine (GEM, 8.0 μg/mL)，Docetaxel (DOC, 0.1 μg/mL) and Pemetrexed (Alimta, 0.90 μg/mL).

**Figure 1:**
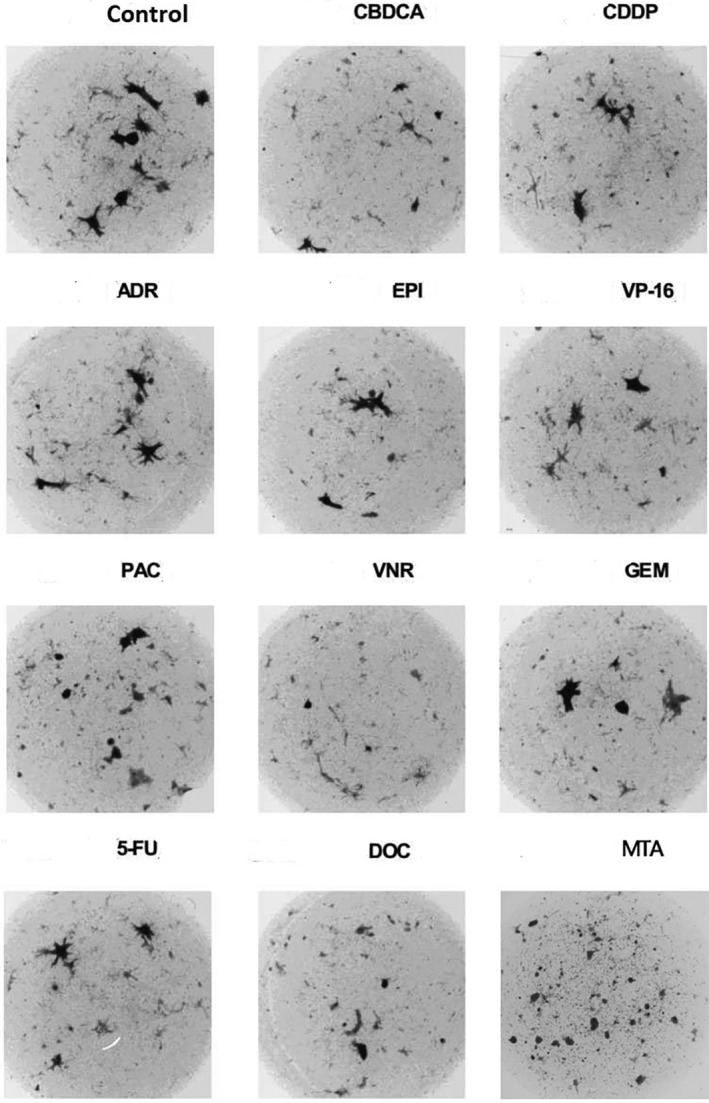
Growth of B2 thymoma cells in collagen gel droplet cultures with different types of chemotherapeutic agents after culture for 5–7 days. By measuring the size of colonies, drug sensitivity was tested. The larger colonies in size were, the less sensitive thymoma cells were to the tested anticancer drugs. CBDCA, 2.0 μg/mL carboplatin; CDDP, 0.2 μg/mL cisplatin; ADR, 0.02 μg/mL adriamycin; EPI, 0.1 μg/mL Epirubicin; VP‐16, 1.0 μg/mL Etoposide; PAC, 1.0 μg/mL Paclitaxel; VNR, 0.01 μg/mL Navelbine; GEM, 8.0 μg/mL Gemcitabine; 5‐FU, 1.0 μg/mL 5‐furuolouracil; DOC, 0.1 μg/mL Docetaxel; MTA, 0.90 μg/mL Pemetrexed.

TETs were pathologically confirmed after surgery. Among these, two cases were type A thymoma, 17 were type AB thymoma, 12 were type B1 thymoma, 44 were type B2 thymoma, 12 were type B3 thymoma, and there were 13 cases with thymic carcinoma. The effect of antitumor agents on cells of TETs was detected by the CD‐DST method. As shown in Fig 1, after five to seven days' growth, the colonies of TET cells were cultured in collagen gel droplets with different antitumor agents and analyzed by the image analysis method (software Primage 1.0.6.3). By measuring the size of colonies, drug sensitivity was tested. The larger the size of the colonies, the higher the growth rates and the lower the drug sensitivities were.

### CD‐DST methods

Each sample was minced finely using a scalpel or razor blade and digested in a cell dispersion enzyme solution (EZ; Kurabo, Japan) for two hours. The dispersed cancer cells were treated with ethylene glycol tetra‐acetic acid (EGTA)‐trypsin and filtered through a 200 μm nylon mesh. The cells were then incubated in a collagen gel‐coated flask (CG‐flask; Kurabo, Japan) containing preculture medium (PCM‐1; Kurabo, Japan) with 10% fetal bovine serum (FBS) at 37°C in 5% CO_2_ overnight. Only the viable cancer cells that adhered to the collagen gel were collected, treated again with EGTA‐trypsin and filtered through a 125 μm nylon mesh.

Type I collagen (Cellmatrix Type CD; Kurabo, Japan), 10X F‐12 medium and reconstruction buffer were added to ice water at a ratio of 8:1:1. The prepared cancer cell suspension was added to the mixed collagen solution with a final density of 2–5 × 10^5^ cells/mL. Three drops of the collagen‐cell mixture (30 μL/drop) were placed in each well of six‐well plates and in a 35 mm dish and left to set at 37°C in a CO_2_ incubator. The final concentration was 3 × 10^3^ cells/droplet. Then one hour later, 3 mL of DF medium containing 10% FBS (DF‐10) was overlaid on each well and 6 mL on the 35 mm dish and incubated in a CO_2_ incubator at 37°C overnight.

At the 0 time control, the drops in the 35 mm dish were stained with neutral red and fixed with 10% formalin and dried. In each well of the six‐well plates, the anticancer drugs were added at final concentrations and incubated for 24 hours. Following the removal of the medium containing the anticancer drugs, each well was rinsed twice, overlaid with serum‐free culture medium (PCM‐2; Kurabo, Japan) and incubated further for seven days. Only 5‐fluorouracil (TS‐1) and Capecitabine (CAP) was left in the culture media for seven days. Control drops were also cultured for seven days without the drugs under the same condition.

After the seven day culture period, neutral red was added to each well at a final concentration of 50 μg/mL, and viable colonies in the droplets were stained for 1–2 hours. Neutral red staining was used to eliminate the interference of fibroblasts. The tumor cells were dyed red by neutral red, while the fibroblasts were not stained or lightly stained. The fibroblasts could then be removed. After that, each droplet was fixed with 10% formalin, washed in water and dried. A video microscope (Kurabo, Japan), grayscale image digitizer (Kurabo, Japan), personal computer and modification of the NIHImage Macro‐program (Primage; Kurabo, Japan) were used to measure and quantify the amount of neutral red dye taken up by the viable cells in the droplets.

When the ratio of control (7‐day culture without drug) to the 0‐time control was >0.8, the case was regarded as assessable. The growth rate of tumor cells was determined by the T/C ratio (T was the image optical density of the chemo‐treated samples on day 7 and C was the image optical density of nontreated controls on day 7). The lower the growth rate of tumor cells was, the greater damage the tested anticancer drugs had on cancer cells, the more sensitive cancer cells were to the tested anticancer drugs.

1. When the growth rate of tumor cells was ≤50%, it showed that individual cancer cells had a high sensitivity to the medicine.
2. When the growth rate of tumor cells was between 50% and 60%, it showed that individual cancer cells were around the boundary of low and high sensitivity (moderate sensitivity).
3. When the growth rate of tumor cells was >60%, it proved that individual cancer cells had a low sensitivity to the medicine.

### Statistical analysis

All analyses were performed with IBM SPSS Statistics 19.0. Continuous variables were expressed as mean ± standard deviation (SD). Discrete variables expressed as mean (range) were compared using paired sample *t*‐tests. The *χ* ^2^ test was used to compare frequencies among different groups. *P*‑values less than 0.05 were considered statistically significant. The image analysis method used in this study was the software Primage 1.0.6.3.

## Results

A total of 36 out of 100 patients with TETs were sensitive to different types of chemotherapeutic agents, while 64 were not sensitive to chemotherapeutic agents as listed in Table 1. There were no significant differences in age, histological type, clinical staging, or association with autoimmune diseases between sensitive and nonsensitive cases. Data information on sensitive cases is listed in Table 2. Among these, six cases were type AB thymoma, six were type B1, 18 were type B2, two were type B3 and there were four cases with thymic carcinoma. One thymoma patient was associated with MG and acute promyelocytic leukemia, one with systemic lupus erythematosus (SLE), one with dermatomyositis, one with MG and mammary cancer, 22 solely with MG, and one with multiautoimmune disorders (including primary adrenocortical hypofunction, optic neuritis, optic nerve degeneration and allergic dermatitis). Among 36 cases sensitive to chemotherapeutic drugs, 18 were solely sensitive to one antitumor agent. WHO histologic subtypes of TETs in relation to the sensitivity to chemotherapeutic agents are shown in Table 3. We found that B1 and B2 types of thymoma were relatively more sensitive to chemotherapeutic agents (6/12 and 18/44, respectively), while sensitivity of B3‐type cases to chemotherapeutic agents was much lower (only 2/12). Among six patients with B1 thymoma sensitive to antitumor agents, four were sensitive to EPI; B2 thymoma was sensitive to a broad spectrum of chemotherapeutic agents (including EPI, PAC, DOC, VNR, GEM and 5FU) as shown in Table 3. For sensitive AB thymoma, four out of six cases were sensitive to EPI. Besides EPI, 2/6 cases were sensitive to CBDCA, PAC, DOC, and VNR, respectively. For sensitive thymic carcinoma, two cases were sensitive to EPI, two to VNR, three to VP‐16, and one to Alimta. Two cases with A‐type thymoma were not sensitive to any antitumor drugs as shown in Table 3.

**Table 2:** Data information of 36 patients sensitive to certain chemotherapeutic agents

Patient Id	Gender	Age	Associated autoimmune disorders/previous malignancy	Size of primary lesion (cm)	Invasion of neighboring organs	Approach of surgery	Clinical resection status	WHO histological type	Chemosensitive agents (survival rate of tumor cells)	Masaoka clinical staging	Metastatis of Lymph Nodes	Distant Metastatis
1	Male	45	MG (IIb)	4.7 × 7.1 × 8.6 cm	Pericardium	Sternotomy	Complete	AB Thymoma	PAC (58.29%)，ALIMTA (59.39%)	III	N0	M0
2	Male	65	Lupus erythematosus	4.7 × 4.1 × 5.3 cm	Left phrenic nerve	VATS	Complete	AB Thymoma	EPI (59.27%)	III	N0	M0
3	Female	63	MG (I)	3.8 × 2.9 × 3.7 cm	No	VATS	Complete	AB Thymoma	EPI (48.75%)	I	N0	M0
4	Male	56	No	3.5 × 2.5 × 1.6 cm	No	VATS	Complete	AB Thymoma	CBDCA (41.22%), VNR (40.75%), DOC (53.17%), EPI (53.22%)	IIa	N0	M0
5	Female	72	No	6.5 × 5.0 × 5.0 cm	No	Sternotomy	Complete	AB Thymoma	CBDCA (48.65%), VNR (45.46%), DOC (58.77%)	IIb	N0	M0
6	Female	47	No	4.8 × 3.9 × 1.5 cm	No	VATS	Complete	AB Thymoma	PAC (52.38%), EPI (58.42%)	I	N0	M0
7	Female	44	MG (IIb)	3.5 × 2.0 × 1.7 cm	Pericardium, innominate vein	Sternotomy	Complete	B1 Thymoma	EPI (56.60%)	III	N0	M0
8	Female	49	No	3.3 × 2.0 × 1.5 cm	No	VATS	Complete	B1 Thymoma	CDDP (50.13%), CBDCA (53.38%), ADR (52.52%), EPI (40.70%), VP‐16 (52.37%)，PAC (17.75%), VNR (59.16%), 5FU (38.45%), GEM (52.53%)，DOC (24.62%)	I	N0	M0
9	Male	44	MG (IIa)	5.5 × 3.5 × 2.7 cm	Right mediastinal pleura	VATS	Complete	B1 Thymoma	EPI (54.00%)	IVA	N0	M1a
10	Male	46	MG (I)	4.0 × 3.5 × 1.5 cm	No	VATS	Complete	B1Thymoma	CDDP (53.57%), CBDCA (57.43%), ADR (57.21%), VP‐16 (57.84%), 5FU (58.88%), GEM (53.75%)，DOC (56.85%)	I	N0	M0
11	Male	33	MG (I)	6.3 × 4.5 × 4 cm	No	VATS	Complete	B1Thymoma	EPI (43.41%)	IIb	N0	M0
12	Male	53	MG (IIb)	5.5 × 3.6 × 5.3 cm	No	VATS	Complete	B1Thymoma	GEM (50.45%)	I	N0	M0
13	Female	50	MG (IIa), Mammary cancer	7.2 × 4.1 × 1.7 cm	Pericardium, right upper lobe, right mediastinal pleura	Sternotomy	Complete	B2Thymoma	EPI (47.58%)	IIb	N0	M0
14	Male	65	No	4.5 × 2.1 × 2.1 cm	No	VATS	Complete	B2Thymoma	EPI (57.54%)	I	N0	M0
15	Male	48	MG (I)	6.3 × 3.4 × 2.3 cm	No	VATS	Complete	B2 Thymoma	VNR (56.32%)	IIb	N0	M0
16	Female	49	No	7.3 × 6.3 × 2.6 cm	Left mediastinal pleura	Sternotomy	Complete	B2 Thymoma	VNR (54.48%)	IIa	N0	M0
17	Female	53	No	12.5 × 9.3 × 5.1 cm	Right phrenic nerve, right mediastinal pleura, pericardium	VATS	Complete	B2 Thymoma	VNR (34.17%)	III	N0	M0
18	Male	64	MG (I)	3.6 × 2.6 × 3.3 cm	Right upper lobe	VATS	Complete	B2 Thymoma	VNR (19.68%)	IIb	N0	M0
19	Male	56	MG (IIb)	2.5 × 1.9 × 2.5 cm	Innominate vein, pericardium, left upper lobe	Sternotomy	Complete	B2 Thymoma	CDDP (54.69%), CBDCA (55.98%), ADR (37.25%), 5FU (33.84%)	III	N0	M0
20	Female	54	MG (I)	2.3 × 2.6 × 1.7 cm	No	Sternotomy	Complete	B2 Thymoma	PAC (54.60%), 5FU (46.29%), GEM (48.36%)，DOC (35.84%)	I	N0	M0
21	Male	32	MG (I)	5.5 × 1.5 × 10 cm	No	VATS	Complete	B2 Thymoma	5FU (48.71%)，PAC (51.34%)，DOC (55.75%), EPI (51.75%)	IIb	N0	M0
22	Female	54	MG (IIb), acute promyelocytic leukemia	3.2 × 1.4 × 2.5 cm	No	VATS	Complete	B2 Thymoma	VNR (17.78%), GEM (23.67%), PAC (39.78%)	IIa	N0	M0
23	Male	38	MG (IIIb)	6.3 × 1.8 × 1.0 cm	Pericardium	VATS	Complete	B2 Thymoma	PAC (51.59%), 5FU (32.49%), GEM (36.47%，DOC (19.70%)，ALIMTA (52.68%)	III	N0	M0
24	Female	53	MG (IIb)	3.5 × 1.5 × 10 cm	No	VATS	Complete	B2 Thymoma	DOC (58.59%)	IIa	N0	M0
25	Male	48	MG (I)	3.3 × 2.7 × 2.0 cm	No	VATS	Complete	B2 Thymoma	5FU (37.72%)，PAC (56.87%)，EPI (53.68%)	I	N0	M0
26	Male	45	MG (IIa)	8.4 × 5.2 × 2.7 cm	No	VATS	Complete	B2 Thymoma	GEM (55.82%)	IIb	N0	M0
27	Female	53	MG (IIIb)	4.7 × 3.5 × 2.5 cm	Pericardium	Sternotomy	Complete	B2 Thymoma	CDDP (35.53%), CBDCA (48.05%), ADR (21.08%), EPI (27.80%), VNR (38.80%), 5FU (47.14%)	III	N0	M0
28	Male	66	Primary adrenocortical hypofunction, optic neuritis, optic nerve degeneration, allergic dermatitis	7.5 × 4.5 × 3.6 cm	Right mediastinal pleura	Sternotomy	Complete	B2 Thymoma	VNR (48.72%), GEM (32.47%), PAC (55.62%)	IVA	N0	M1a
29	Male	54	MG (I)	4.9 × 3.2 × 6.0 cm	Pericardium, right upper lobe, right middle lobe, right mediastinal pleura	VATS	Complete	B2 Thymoma	DOC (56.49%)	IIb	N0	M0
30	Female	66	No	5.4 × 4.0 × 5.2 cm	No	VATS	Complete	B2 Thymoma	PAC (55.56%)	I	N0	M0
31	Female	55	MG (I)	5.2 × 9.5 × 11.3 cm	Innominate vein, pericardium, left upper lobe	Sternotomy	Incomplete	B3 Thymoma	GEM (51.52%)，DOC (54.47%)	IVA	N0	M1a
32	Female	43	MG (I)	11 × 10.5 × 5.5 cm	Innominate vein, pericardium, right upper lobe, right mediastinal pleura, superior vena cava	Sternotomy	Complete	B3 Thymoma	EPI (50.59%), GEM (55.79%)，DOC (53.41%)	IVA	N0	M1a
33	Female	54	No	3.6 × 2.5 × 2.2 cm	No	VATS	Complete	Thymic Carcinoma	EPI (53.01%), VP‐16 (59.15%)	I	N0	M0
34	Male	61	Dermatomyositis	15.2 × 6.8 × 4.7 cm	Innominate vein, pericardium, left upper lobe, superior vena cava	Sternotomy	Complete	Thymic Carcinoma	EPI (50.07%), VP‐16 (56.65%)	III	N0	M0
35	Male	49	No	7.4 × 4.7 × 6.6 cm	Innominate vein, pericardium, right upper lobe, right mediastinal pleura, superior vena cava	Sternotomy	Complete	Thymic Carcinoma	VNR (55.46%), VP‐16 (58.89%)	III	N0	M0
36	Female	69	MG (IIb)	7.4 × 3.7 × 9 cm	Innominate vein, superior vena cava	Sternotomy	Complete	Thymic Carcinoma	VNR (58.50%)	III	N0	M0

**Table 3:** Drug sensitivities of different World Health Organization histologic subtypes of thymoma and thymic carcinoma

	Number	Sensitive cases	Sensitive chemotherapeutic agents	Percentage of sensitive cases
A	2	0	0	0
AB	17	6	Two cases were sensitive to CBDCA; four to EPI; two to PAC; two to DOC; two to VNR	35.3%
B1	12	6	Two cases were sensitive to CBDCA; two to CDDP; four to EPI; one to PAC; two to DOC; one to VNR; three to GEM; two to 5FU; two to VP‐16	50%
B2	44	18	Two cases were sensitive to CBDCA; two to CDDP; five to EPI; seven to PAC; five to DOC; seven to VNR; five to GEM; six to 5FU	40.9%
B3	12	2	One case was sensitive to EPI; two to DOC; two to GEM	16.7%
Thymic carcinoma	13	4	Two cases were sensitive to EPI; two to VNR; three to VP‐16; one to Alimta	30.8%

As shown in Table 4, among 11 chemotherapeutic agents tested in our study, the sensitivity to EPI was the highest (16%), while Alimta had the lowest sensitivity. No patients with thymoma were sensitive to Alimta. Only one patient with thymic carcinoma was moderately sensitive to Alimta. Besides EPI, the sensitivities to PAC, DOC, VNR and GEM were over 10%. Our data demonstrated that CDDP, ADR, and 5‐FU were effective medicines for some patients with type B1 or B2 thymoma; CBDCA, PAC, VNR and GEM were effective for some cases with types AB, B1 or B2 thymoma; VP‐16 was effective for some patients with B1‐type thymoma or thymic carcinoma; EPI could be useful for most subtypes of thymoma and thymic carcinoma except type A thymoma; and DOC could be used for most subtypes of thymoma except type A thymoma.

**Table 4:** Effectiveness of certain chemotherapeutic agents to treat TETs

			Subtypes of thymic malignant tumors						
	High‐sensitivity	Moderate‐sensitivity	A	AB	B1	B2	B3	Thymic carcinoma	Number of sensitive cases (%)
CDDP	1	3	0	0	2	2	0	0	4 (4%)
CBDCA	3	3	0	2	2	2	0	0	6 (6%)
ADR	2	2	0	0	2	2	0	0	4 (4%)
EPI	5	11	0	4	4	5	1	2	16 (16%)
VP‐16	0	5	0	0	2	0	0	3	5 (5%)
PAC	2	8	0	2	1	7	0	0	10 (10%)
VNR	7	5	0	2	1	7	0	2	12 (12%)
5FU	7	1	0	0	2	6	0	0	8 (8%)
GEM	4	6	0	3	5	2	0	0	10 (10%)
DOC	3	8	0	2	2	5	2	0	11 (11%)
ALIMTA	0	1	0	0	0	0	0	1	1 (1%)

## Discussion

TETs, such as thymoma and thymic carcinoma, are peculiar epithelial neoplasms located at the anterior mediastinum. Some may show aggressive clinical behavior, while the majority demonstrate an indolent growth pattern.^15^, ^16^ Complete resection is generally regarded as the most effective treatment for patients with malignant tumors, provided that the tumors are resectable. The development of targeted therapies has been delayed by the insufficient characterization of the genetic abnormalities of thymic malignant tumors.^17^ Nonresectable and metastatic thymic malignant tumors are candidates for chemotherapy. Efficacy of chemotherapy varies between reports. Some studies have reported that chemotherapy achieved tumor responses in 60%–80% of patients,^18^, ^19^ while some clinical data demonstrated that the result of chemotherapy was not fully satisfactory in thymic malignant tumors.^20^, ^21^

To the best of our knowledge, our current work is the first systemic evaluation of sensitivity of all pathological types of TET cells to common chemotherapeutics using the CD‐DST method. CD‐DST has been developed as an evaluation system of effective anticancer drugs. This culture system models the 3‐D growth of cancer cells and has been proven to show high predictive accuracy for clinical responses to anticancer drugs.^22^, ^23^

A number of anticancer drugs have been reported to treat thymic malignant tumors.^9^, ^24^, ^25^, ^26^ In our current study, we tested 11 kinds of anticancer drugs, which have been reported in former studies to be possibly effective to treat thymoma or thymic carcinoma. Among 100 cases with TETs, 36% were sensitive towards different chemotherapeutic agents. Our data showed that age, histological type, clinical staging, or association with autoimmune diseases were not significantly associated with drug responsiveness.

In our study, there was no single drug that showed a high clinical response. Indeed, although the sensitivity of TETs to EPI was the highest, it was only 16%. In addition to EPI, the sensitivities to PAC, DOC, VNR and GEM were over 10%, echoing conclusions in earlier literature that the efficacy of anticancer drugs in the treatment of TETs is not remarkable.^20^, ^21^ Possibly due to the indolent growth pattern of thymic malignant tumors, the sensitivity of TET cells to common chemotherapeutics is dramatically different from other types of cancer. For example, cisplatin sensitivity was observed in 4% of cases in this study, whereas the response rates to single‐agent cisplatin reported in lung cancer are approximately 20%–30%. The combination of anticancer drugs to treat thymoma or thymic carcinoma might produce a relatively higher percentage of clinical responses, as reported in previous studies.^24^, ^25^, ^26^

Of note, EPI seemed to be the best choice of B1 thymoma, while for patients with B2 thymoma, EPI, PAC, DOC, VNR, GEM and 5FU might be good choices (as shown in Table 3). For sensitive AB thymoma, four out of six cases were sensitive to EPI. In addition to EPI, 2/6 cases were sensitive to CBDCA, PAC, DOC, and VNR, respectively. These results are worth taking into consideration in setting up clinical guidance for patients with TETs. In our study, it should be noted that Alimta had the lowest sensitivity and no patients with thymoma were sensitive to Alimta, which is in drastic contrast with current dogma. Our work therefore suggests a patient care practice: screening with a CD‐DST culture system to determine the drugs for treating patients with nonresectable and metastatic thymic malignant tumors.

In conclusion, the successful selection of chemotherapy is necessary for TETs, and information on in vitro drug sensitivity or resistance may be valuable to guide individual chemotherapy. An important aspect of our work was to shed light on the effectiveness of chemotherapy for TETs and provide important clues for choosing antitumor drugs with relatively high drug sensitivity to certain type of TETs in advance.

## Disclosure

The authors declare no conflict of interest.
